# Philadelphia Hospital

**Published:** 1839-01-05

**Authors:** 


### PHILADELPHIA HOSPITAL.

### BURNS—STAPHYLOMA—CANCROIDES, ETC.

*Saturday, December 22d.*—Dr. Gibson commenced by exhibiting to the class the several patients upon whom he had at previous meetings operated. The operation for entropium on both eyelids, had been, he stated, attended with success, and the man declared himself materially benefited. The cases of phymosis and paraphymosis were again shown, and appeared all to be doing very well—either entirely relieved, or rapidly progressing to a happy termination. The case of hydrocele, which had been operated on by the seton, was next introduced. This man was also doing well. Slight suppuration had occurred in the track of the seton. This, Dr. G. remarked, was generally the case,—and is not to be looked on as a bad omen, but rather as a good one, for a more certain cure follows. Here is the man, continued Dr. G., from whose neck I removed last Saturday the adipose tumour. The wound is now granulating kindly. He has suffered, however, so much pain in it and the adjacent parts, as not to permit the edges to be drawn together. It is now eight days since the operation. I will examine into the condition of the ligatures; and, on account of the number of vessels which sprang during its excision, there are a good many. You see they all come away readily. I will now draw the lips of the wound fairly together, for it must not be permitted to gape any longer, as its healing would be retarded. This I have now done, and in a short time you will find it healed. The boy whose great-toe I removed last week for incurable deformity, arising from a burn, is doing well; the wound has nearly healed; and he will walk without impediment, though it will yet require some effort to restore the foot to its natural position. This may be done, perhaps, by bandages and splints, or by machinery, or possibly it may be necessary to make a slight cut through the *sound* skin, either above or below the cicatrix of the burn. The next case I shall present to your notice is a very extensive burn, which occurred three weeks since. I have not shown it before, on account of the danger that might have attended the exposure; for if a chill had been induced, the patient's chance of recovery would have been diminished. This is a common accident at this season, and much more common among females than males, from the circumstance of their clothing being more combustible than men's. Extensive burns, although superficial, are often followed by more serious consequences than severe burns which are more limited.

Every surgical writer follows some method of classification in treating of burns. The one I prefer is that of Pearson. 1st. Superficial burns, producing inflammation of the cutaneous system, terminating, generally, in resolution. 2d. Deep-seated burns, in which the true skin is involved, and ulceration occurs to a greater or less extent; and, 3d, where the vitality and texture of the part is completely destroyed and charred to a cinder. When a burn occurs on the body, a chill generally takes place, followed most frequently by altratic symptoms, such as great oppression, and difficulty of breathing. Now, observe the extent of this burn—the whole side, and greater part of the back are involved. It is now presented to you in a state of ulceration,—the constitutional symptoms have subsided, the immediate danger has passed, and the patient is doing very well. In five or six weeks you will find it nearly skinned over, except minute granulating surfaces, scattered here and there, like little islands. The best treatment in the latter stages of a burn is a stimulating one,—new skin must be produced, and the process of cicatrization hastened. There are several modes of treating recent burns. When the skin is not broken, *raw cotton* is the best. In a case like this cotton would be injurious, inasmuch as its spicula would adhere to the granulations, and produce irritation. Its discovery in the treatment of burns was accidental. A lady of Maryland happened to be carding cotton in an adjoining room, and hearing the cries of her child, who had been severely burnt, wrapped it up in the cotton. The child's sufferings were very soon mitigated, and it speedily recovered. The particulars of the case were published by Dr. Dallam, and from that period cotton has been held in high esteem in the treatment of superficial burns. In the third, or carbunculous variety of burn, stimulating applications are the most appropriate,—and of these the turpentines are the best. In the first variety of burn, where the cuticle is elevated, the vesicles should be punctured. Some persons I have known in such cases peel it off; this ought not to be done; it exposes the true skin, and does mischief. In these cases the linseed oil and lime water is the best topical application; but the applications during the progress of the cure, require to be frequently changed.

Secondary affections, of a very troublesome character, frequently follow burns, such as contractions and adhesions. I here show you what was once a very extensive burn, not so large, indeed, as the one you have just seen, but still much larger than from the present appearance you would be likely to suppose. It is ten months since it happened, and it is not healed entirely yet; and some time will elapse before cicatrization is perfect. The arm, you observe, cannot be elevated, but is bound down to the side by the adhesion of the skin to the pectoral muscle. By the time the sore is healed, it will be completely pinned down. Similar cases are constantly occurring, from the carelessness or ignorance of practitioners. The eye, neck, hands, or feet, are generally the sufferers. The plan recommended by Mr. Henry Earle, is to remove the entire cicatrix. This is not always practicable, as in the present case, where it is so extensive. I might perform an operation for the relief of this man, by dividing the cicatrix; but, from its great extent, I am inclined to believe that no permanent benefit would result, inasmuch as there would be strong tendency to subsequent contraction. As the patient is anxious to be relieved, I may, at a future period, cut through about one third of the pectoral muscle, trusting to the remaining portion having sufficient power left to answer its original purpose.

This man I next show is suffering from a very serious affection of the eyeball, called *staphyloma*, from its resemblance to a grape, or bunch of grapes,—for sometimes there is one tumour, at other times more. In this disease, the transparency of the cornea becomes destroyed, and it projects in a conical form beyond the lids as a pearl-coloured tumour, sometimes smooth, sometimes irregular. From the exposure of the eyeball from the protuberance, it is continually catching extraneous bodies, which produce a vast deal of irritation. This man, during the excessive heat of last summer, after working all day in the harvest field, took a cold bath, while heated, in the Schuylkill. Violent inflammation of the eye resulted, and finally this caused the condition of the staphyloma. No topical application that we can make will restore the transparency of the cornea, and enable him to regain the sight which is lost. The only thing left for us to do, is to relieve the man, and improve the appearance of the eye, by an operation. This he himself is anxious for, as he thinks the other eye is becoming affected. This apprehension may be well grounded. I shall pass the cataract knife through the cornea, and make a section of it; I shall then lay hold of the flap with the forceps, and remove it. The crystalline lens and vitreous humour will be evacuated, and the eye will collapse. Were I simply to puncture the tumour, the wound would soon close up, and the same condition would speedily recur. [Dr. G. operated, and with the effect just stated.]

Here is a case which, from its appearance, you would probably pronounce to be the cicatrix of a burn, and, indeed, it bears a very strong resemblance to one. It is, however, a disease called *cancroides*, first accurately described by Alibert in his valuable work on the diseases of the skin. In this country it is rarely met with, although I have seen it several times, and always among the negroes. I have seen it hanging from the lobe of the ear like a prickly pear, and in these cases the patients had all been in the habit of wearing brass ear-rings, and to that circumstance attributed the disease. Some years ago I removed a tumour of this kind from the neck and ear of a man in this hospital; a severe chill followed the operation, succeeded by fever and high cerebral excitement, and death. On examination, the membranes of the brain were found extensively inflamed. It is true that the man was of bad constitution and of intemperate habits, but still there are strong reasons for believing that the operation produced more irritation than under similar circumstances could have been anticipated, and probably was the immediate cause of death. Alibert inclines to the opinion that it is a constitutional, and not a local affection, and that it will return after an operation. I have removed tumours of this description six or seven times; some I have found to return, and others not. Since, however, the particular case I have spoken of, I have been afraid to meddle with them. As this is productive of great inconvenience to the patient, by distorting and closing the right eyelids and contracting the muscles of the face, I will give her case further consideration, and endeavour to think of some means of affording her relief.

